# Solanaceae glycoalkaloids: α-solanine and α-chaconine modify the cardioinhibitory activity of verapamil

**DOI:** 10.1080/13880209.2022.2094966

**Published:** 2022-07-10

**Authors:** Szymon Chowański, Magdalena Winkiel, Monika Szymczak-Cendlak, Paweł Marciniak, Dominika Mańczak, Karolina Walkowiak-Nowicka, Marta Spochacz, Sabino A. Bufo, Laura Scrano, Zbigniew Adamski

**Affiliations:** a Department of Animal Physiology and Developmental Biology, Faculty of Biology, Adam Mickiewicz University, Poznań, Poland; b Laboratory of Electron and Confocal Microscopy, Faculty of Biology, Adam Mickiewicz University, Poznań, Poland; c Department of Sciences, University of Basilicata, Potenza, Italy; d Department of Geography, Environmental Management and Energy Studies, University of Johannesburg, Johannesburg, South Africa; e Department of European Culture, University of Basilicata, Matera, Italy

**Keywords:** L-type calcium channels, insects, myocardium, heart

## Abstract

**Context:**

Solanaceae glycoalkaloids (SGAs) possess cardiomodulatory activity.

**Objective:**

This study investigated the potential interaction between verapamil and glycoalkaloids.

**Material and methods:**

The cardioactivity of verapamil and glycoalkaloids (α-solanine and α-chaconine) was tested in adult beetle (*Tenebrio molitor*) myocardium *in vitro* using microdensitometric methods. The myocardium was treated with pure substances and mixtures of verapamil and glycoalkaloids for 9 min with saline as a control. Two experimental variants were used: simultaneous application of verapamil and glycoalkaloids or preincubation of the myocardium with one of the compounds followed by perfusion with a verapamil solution. We used 9 × 10^−6–5^ × 10^−5^ M and 10^−9^–10^−5^ M concentration for verapamil and glycoalkaloids, respectively.

**Results:**

Verapamil, α-solanine and α-chaconine showed cardioinhibitory activity with IC_50_ values equal to 1.69 × 10^−5^, 1.88 × 10^−7^ and 7.48 × 10^−7^ M, respectively. When the glycoalkaloids were applied simultaneously with verapamil, an antagonistic effect was observed with a decrease in the maximal inhibitory effect and prolongation of t_50_ and the recovery time characteristic of verapamil. We also confirmed the expression of two transcript forms of the gene that encodes the α1 subunit of L-type calcium channels in the myocardium and brain with equal transcription levels of both forms in the myocardium and significant domination of the shorter form in the brain of the insect species tested.

**Discussion and conclusions:**

The results show that attention to the composition of the daily diet during therapy with various drugs is particularly important. In subsequent studies, the nature of interaction between verapamil and SGAs on the molecular level should be checked, and whether this interaction decreases the efficiency of cardiovascular therapy with verapamil in humans.

## Introduction

Steroidal glycoalkaloids are organic compounds with a ring structure, usually of plant origin. They are heterocyclic bases that contain a nitrogen atom and consist of a sugar moiety and an aglycone. The hydrophobic and nonpolar part of the aglycone is a steroidal structure to which carbohydrate moieties are attached at the 3-OH position. The polar, water-soluble sugar part contains three or four monosaccharide molecules of D-glucose, D-galactose, D-xylose, and L-rhamnose in various combinations (Friedman 2004, 2006; Nepal and Stine 2019). These compounds are produced by plants as secondary metabolites and used in protection against herbivores and as antipathogenic agents during fungal or bacterial infection (Roddick 1996; Chowański et al. 2016). They show properties such as analgesic, anti-inflammatory, antitumor, cardiovascular, and antioxidant effects, or activity that prevents muscle wasting. Thus, their high biological activity enables many of them to be used as pharmacological agents (Niño et al. 2009; Friedman 2013; Kolińska et al. 2016; Jan et al. 2017; Dey et al. 2019; Ebert et al. 2019; Morais et al. 2020).

Among the representatives of steroidal glycoalkaloids are alkaloids produced by the *Solanaceae* plant family, such as α-solanine, α-chaconine, α-solamargine, α-solasonine, and α-tomatine. These compounds are commonly found in many popular food products containing tomatoes, potatoes, or eggplants (Friedman 2004). Solanaceae glycoalkaloids (SGAs) show high biological activity. For vertebrates, SGAs were shown to decrease respiratory activity and blood pressure and to cause bradycardia and haemolysis; at high concentrations they have hepatotoxic activity and can act as irritating agents within the digestive tract (Friedman 2006). Their teratogenic activity was also confirmed (Blankemeyer et al. 1998).

In addition, SGAs are potent inhibitors of enzymes involved in the breakdown of the neurotransmitter acetylcholine. Blocking acetylcholinesterase (AChE) and butyrylcholinesterase (BuChE) causes the accumulation of acetylcholine in the central nervous system. This could result, e.g., in impaired balance and motor coordination, shortness of breath and increased heart rate (Friedman 2006). SGAs also show high activity in insects. For example, they have substantial effects on the activity of the insect myocardium, affecting not only the frequency and force of insect heart contraction but also the duration of circadian phases of heart activity (Ventrella et al. 2015; Marciniak et al. 2019). Furthermore, SGAs affect the structure of fat body cells and the midgut and change the carbohydrate profile of insect haemolymph (Spochacz et al. 2018, 2020, 2021). Therefore, due to similar effects observed in insects and mammals, insects can serve as good models for testing the effects of these substances on mammals, including humans.

The biological activity of SGAs is probably related to the inhibition of AChE and the disturbance of cellular homeostasis by calcium, potassium, and sodium ions. It was shown that these ions could change the ionic concentration across the cell membrane and transepithelial transport of ions (Michalska et al. 1985; Toyoda et al. 1991; Blankemeyer et al. 1995, 1997, 1998). Moreover, SGAs may interact with cholesterol and form tubular and spherical structures in cell membranes, changing their permeability (Keukens et al. 1995, 1996). Finally, the activities mentioned above affect the cell membrane potential (Blankemeyer et al. 1998), thereby changing the activity of excitable cells, such as neurons and muscle cells.

Calcium ions play a crucial role in muscle contractions, and therefore, L-type calcium channels that move Ca^2+^ ions inward and trigger calcium release from the sarcoplasmic reticulum by activating the ryanodine receptor 2 (Striessnig et al. 2014) are just as important. Dysregulation of L-type Ca^2+^ channels is the basis of numerous cardiac disorders; therefore, they are also a common target in various therapies for cardiovascular diseases. L-type Ca^2+^ channel blockers, such as verapamil, are commonly used to treat hypertension, myocardial ischaemia, and arrhythmias (Limpitikul et al. 2018). The so-called α1 subunit forms the core of voltage-sensitive L-type Ca^2+^ channels. It associates with other subunits (β, α2δ, γ) to form heterooligomeric complexes. The β and α2/δ subunits are tightly but not covalently bound to the α1 subunit and modulate the biophysical properties and trafficking of the α1 subunit to the membrane (Bodi et al. 2005). The presence of L-type Ca^2+^ channels were also confirmed in the myocardium of *Drosophila melanogaster* (Limpitikul et al. 2018) and *Musca domestica* (Grabner et al. 1994). This tissue builds the dorsal vessel of the insect, traditionally called the heart. Even if not anatomically, the insect heart functionally and developmentally resembles the embryonic vertebrate heart. Thus, it offers an attractive alternative for studies conducted on mammals. Furthermore, many analyses can be performed *in vivo* without the need to sacrifice the test animal (Limpitikul et al. 2018).

Verapamil is a prototypical phenylalkylamine and was the first calcium channel blocker used clinically. It tonically blocks L-type channels with micromolar affinity (DrugBank 2021). Based on the relative specificity of the L-type Ca^2+^ channel antagonist, verapamil blocks conduction, especially in sinus nodal-like cells. Consequently, it changes the spiking rhythmicity and electrical propagation, e.g., in embryonic stem cell-derived cardiomyocytes (Reppel et al. 2007), and thus, decreases myocardial contractility with negative inotropic and chronotropic effects (Kurola et al. 2010). Due to its properties, it is commonly used as an antiarrhythmic and vasodilating medication.

The interactions between many bioactive compounds are widespread, both between drugs and nondrug substances (Koziolek et al. 2019). These interactions might change their activity, including the intensity of the expected effects and the type of evoked effects. In recent years, extensive efforts have been made to elucidate the mechanisms that drive pharmacokinetic food–drug interactions: both, those occurring in the gastrointestinal tract and those taking place in the human body after absorption (Koziolek et al. 2019). Considering that verapamil is a common cardiovascular drug, SGAs are present in many popular food products and can be ingested in reasonably large quantities, and that both have cardioactive potential, we wondered whether SGAs can modulate the activity of verapamil. To explore this hypothesis, we performed experiments on semi-isolated insect hearts treated simultaneously with glycoalkaloids and verapamil and analysed the pharmacokinetic parameters of their activity.

## Materials and methods

### Insects

Adult *Tenebrio molitor* beetle insects were obtained from a culture maintained at the Department of Animal Physiology and Developmental Biology of Adam Mickiewicz University in Poznań. The insects were kept as previously described by Rosiński et al. (1979) under constant conditions of temperature 26 ± 1 °C, relative humidity 60 ± 5% and photoperiod 8:16 h of light to dark in containers filled with flour. Additionally, fresh lettuce leaves and carrot slices were provided twice a week.

### Compounds

The pure glycoalkaloids: α-chaconine (≥95%) and α-solanine (≥95%) were purchased from Lab Service Analytica (Anzola dell'Emilia, Italy), while (±)-verapamil hydrochloride (≥99%) was purchased from Sigma-Aldrich (St. Louis, MO). Chemicals were dissolved in appropriate saline for beetles (274 mM NaCl, 19 mM KCl, 9 mM CaCl_2_, 5 mM glucose and 5 mM HEPES, pH 7.0) (Pacholska-Bogalska et al. 2018) to obtain stock solutions at concentrations of 10^−3^ M for glycoalkaloids and 10^−4^ M for verapamil. The solutions were then kept at −20 °C and the tested dilutions were prepared prior to the experiments. First, different concentrations of verapamil were tested within the range of 9 × 10^−6^ to 5 × 10^−5^ M. The concentration that caused a 75% decrease in heart contraction frequency (EC_75_) was chosen for the following experiments to examine the interactions between verapamil and glycoalkaloids. In the text, ver + sol indicates the mixture of verapamil and α-solanine, while ver + chac indicates the combination of verapamil and α-chaconine. Similarly, various concentrations of glycoalkaloids were tested (from 10^−9^ to 10^−5^ M), and the concentration that caused the strongest cardioinhibition was used in the ensuing experiments.

### Preparation of semi-isolated heart

For all experiments, only 4-week-old adult insects were used, and the heart preparations were prepared as described previously by Chowański and Rosiński (2017); Pacholska-Bogalska et al. (2018). First, the insects were anaesthetized with CO_2_ for 8 min. Then, after decapitation, the wings and legs were removed. For the next step, only the abdomen was used. With microsurgical scissors and tweezers, the ventral side of the cuticle was removed. The preparations were washed with saline and the visceral organs (fat body, gut, Malpighian tubules and reproductive system) were removed. Subsequently, the semi-isolated heart was placed in saline and left for 10 min to restore the normal rhythm of heart contraction. After checking the condition of hearts, they were placed in the incubation chamber of the microdensitometer.

### *In vitro* heart bioassay

A microdensitometric method, described previously (Marciniak et al. 2008; Chowański and Rosiński 2017; Chowański et al. 2017; Pacholska-Bogalska et al. 2018), was used to analyse the cardiotropic effects of the compounds tested. It allows the measurement of the heart contraction frequency of a semi-isolated heart in insects. Briefly, this method uses a light beam that passes through the myocardium. The density of the tissue changes temporally during the heart cycle, increasing during contraction and decreasing during relaxation. Thus, the amount of light that passes through the myocardium also changes. The intensity of the light beam transmitted through the myocardium is recorded by photodiodes and converted into an electrical signal presented as a cardiomyogram. The signal is registered and converted with LARWA software designed in our department. The experiments were conducted on a semi-isolated heart, the preparation of which is described above. A semi-isolated heart was placed in an incubation chamber and perfused with saline at a flow rate of 300 μL/min. After 5 min of preincubation, registration was started, and the signal was recorded for 22 min. During that time, the preparation was perfused with saline or various solutions of the tested compounds.

### Application pattern of tested compounds

**Figure 1.**
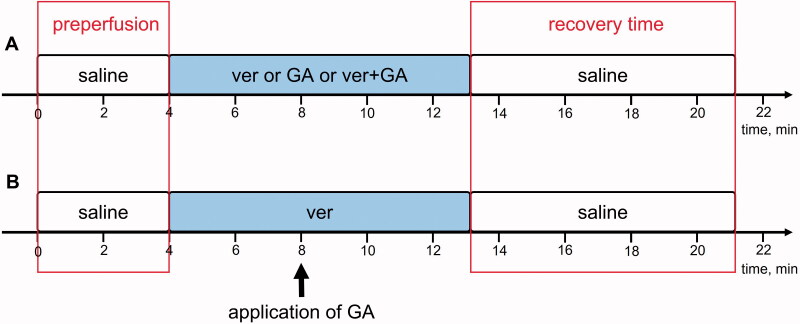
Time scheme of experimental approaches used in the experiments. The blocks correspond to the time when a heart was perfused with different solutions. (A) Experiments with continuous perfusion; (B) experiments with pulse application of tested glycoalkaloids. Ver: verapamil; GA: glycoalkaloids.

We used two experimental variants to test the interaction between verapamil and glycoalkaloids. In variant A, hearts were constantly perfused with a solution of verapamil, a solution of one of the tested glycoalkaloids, or a mixture of verapamil and one of the tested glycoalkaloids (Figure 1(A)). In variant B, the tested glycoalkaloids were applied once with a microsyringe (Hamilton, AL) in a volume of 10 μL onto a semi-isolated heart during continuous perfusion with verapamil (Figure 1(B)). The glycoalkaloids were injected into an application port located on the tube, providing a solution to the incubation chamber. In the text, this variant is called a pulse application. The time pattern of the experiment is presented in Figure 1.

### Analysed parameters

We analysed several parameters to determine the effects of the compounds tested on heart activity and their interaction. As a base, the heart contraction frequency calculated with ANALIZA software (designed in our department) was used (Marciniak et al. 2008; Chowański and Rosiński 2017). Next, we counted the percentage of changes in heart contraction frequency, with the frequency in the first minute of the records used as a reference value. Moreover, to analyse the dynamics of changes in heart rate, we determined the slope coefficient of the curve of the contraction frequency curve (*a*). It was calculated using formula (1):

(1) $$a=\frac{y_{2}-\;y_{1}}{x_{2}-\;x_{1}},$$

where y_2_ and y_1_ are the percentage changes in heart contraction frequency in the exact minute and the previous minute, respectively, and x_2_ and x_1_ are the time values for the respective values of y_2_ and y_1_. Additionally, we determined the time t_50_ (time after which 50% of the maximum change is observed) and the recovery time RT_50_ (time after which the analysed parameter recovers 50% of the initial value). Both parameters were determined with nonlinear analysis in GraphPad Prism 9 software ((La Jolla, CA) (Department of Animal Physiology and Developmental Biology, AMU licence ID: 3E51CFFB054). We also analysed the types of interaction between verapamil and SGAs (additive, antagonistic or synergistic) using two formulas (2) (Berenbaum 1989; Ntalli et al. 2016) and (3):

(2) $${\mathrm{TI}}_{\mathrm{mix}}=\frac{E_{\mathrm{ver}+\mathrm{SGAs}}\;}{E_{\mathrm{ver}}+\;E_{\mathrm{SGAs}}}$$

and

(3) $${\mathrm{TU}}_{\mathrm{mix}}=\frac{E_{\mathrm{ver}+\mathrm{SGAs}}}{E_{\mathrm{ver}}}+\frac{E_{\mathrm{ver}+\mathrm{SGAs}}}{E_{\mathrm{SGAs}}},$$

where TI_mix_ = toxic index, TU_mix_ = toxic units, E_ver+SGAs_ = effect caused by the mixture of verapamil and one of the SGAs, E_ver_ = effect caused by verapamil, and E_SGAs_ = effect caused by a single SGA.

### Identification of L-type calcium channel transcripts

The transcripts of the α1-subunit of the L-type calcium channel were determined in selected tissues of 4-day-old adults with RT-PCR performed according to a modification of the method described by Marone et al. (2001) and used by our group previously (Marciniak et al. 2020; Słocińska et al. 2020) and was done as follows. Tissues (myocardium, brain) were collected from at least ten insects, while whole body samples were collected from three individuals. After dissection, the samples were homogenized in RNA lysis buffer (Zymo Research, Irvine, CA) using a pellet homogenizer. The tissues were then immediately frozen in liquid nitrogen and stored at −80 °C. For RNA extraction, a Quick-RNA Mini Prep kit (Zymo Research) was used according to the manufacturer’s instructions. RNA concentrations and quality were checked with a Synergy H1 Hybrid MultiMode Microplate Reader (BioTek, Winooski, VT). RNA (300 ng) was used as a template to perform reverse transcription with the RevertAid reverse transcriptase kit (Thermo-Fisher, Waltham, MA) according to the manufacturer’s protocol and with the following concentrations: template RNA quantity 300 ng, oligo(dT)_18_ 5 μM, 1 × reaction buffer, Thermo Scientific RiboLock RNase Inhibitor 1 U/μL, dNTP 1 mM, ReverAid Reverse Transcriptase 10 U/μL.

To obtain a sequence of α1 subunits of L-type calcium channels, the transcriptome of the brain and retrocerebral complex of *T. molitor* (SRX7959730 and SRX7805297, BioProject PRJNA608239) was searched using the tblastn algorithm with *T. castaneum* α1 subunits of the L-type calcium channel sequence (NP_001159382.1). The primer pair was designed using Primer3 software, which is part of the Geneious version 9.1.8 package (Untergasser et al. 2012), yielding the following sequence: forward: 5′-TGTTCGACTGTCTAGTGAACTCA-3′ and reverse: 5′-CGTGATGATGATCAAGGCTACG-3′. The size of the RT-PCR product was 448 bp. The primers were synthesized by the Institute of Biochemistry and Biophysics of the Polish Academy of Science (Warsaw, Poland). PCR was carried out in a 10 μL reaction volume, and the final mixture contained 1 μM primers, 200 μM dNTPs, 1× PCR buffer and 1 U/25 μL DreamTaq Polymerase mixture (Thermo Scientific). After PCR, the products were analysed by electrophoresis using a 1.5% TAE agarose gel stained with ethidium bromide and the bands were visualized with ChemiDoc^™^ Touch (Bio-Rad, Hercules, CA). To confirm our results, we isolated the PCR reaction products (separate band) from the agarose gel using the ZymocleanTM gel DNA Recovery Kit (Zymo Research) according to the manufacturer’s instructions. The DNA fragments purified from the agarose gel were then sequenced in the Sequencing and Molecular Biology Laboratory of the Faculty of Biology at Adam Mickiewicz University in Poznań. To confirm that the obtained PCR product is a coding sequence for the α1-subunit of the L-type calcium channel gene, the obtained results were compared with sequences deposited in public databases using the Geneious version 9.1.8 package and the BLAST programs (http://blast.ncbi.nlm.nih.gov/blast.cgi). Furthermore, the ‘no template control’ and ‘no RT control’ reactions were included in the analysis to ensure the absence of foreign or genomic DNA contamination. In each analysis, the Rpl16a gene was used as a positive control.

### Statistical analysis

All data obtained in the experiments were statistically analysed with GraphPad Prism 9 software (La Jolla, CA). At the beginning of the analysis, the normality of the distribution and the homogeneity of the variance was checked with Shapiro–Wilk and Levene’s tests, respectively. One-way ANOVA for statistical comparison of groups with normal distribution, and for nonparametric data, the Kruskal–Wallis test was used. Furthermore, we also performed a two-way ANOVA with Dunn’s multicomparison test. The data presented are the mean values of the parameter ± SD. For each variant, 15–16 repetitions were performed.

## Results

### Effect of verapamil on heart action

**Figure 2.**
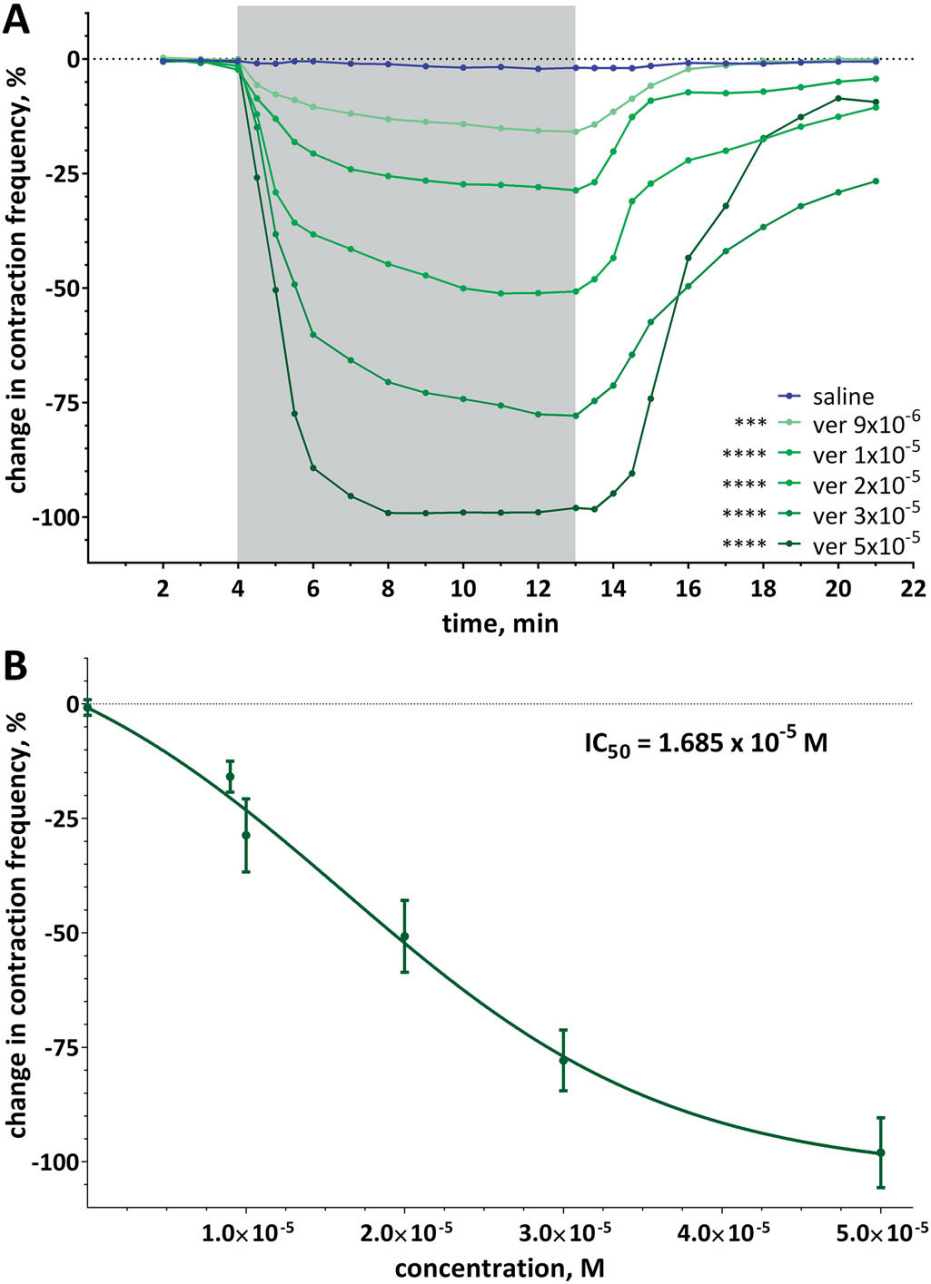
(A) Changes in contraction frequency of semi-isolated heart of the beetle *T. molitor*, perfused with different concentrations of verapamil, in time. Asterisks indicate statistically significant differences in comparison to control (saline) if *p* ≤ 0.0001 (****) and *p*≤ 0.001 (***); *n* = 15; two-way ANOVA. The SD values are not shown to keep the clarity of the diagram. The grey and white background indicates when the hearts were perfused with verapamil solutions or saline, respectively. (B) The changes of heart contraction frequency in the last min of perfusion with different concentrations of verapamil indicating IC_50_ concentration.

A semi-isolated heart placed in an incubation chamber of the microdensitometer was able to work uninterruptedly for a minimum of 5–6 h with saline perfusion. During the registration of its control action, for 22 min, the heart contraction frequency did not change by more than ± 3% (Figure 2(A)). When saline was replaced with verapamil solutions, a decrease in heart frequency was observed, and the changes depended on the concentration. The highest concentration of verapamil tested (5 × 10^−5^ M) caused an arrest of myocardial activity in almost all preparations after a mean time of 1.70 ± 0.77 min, and the calculated t_50_ was 1.04 ± 0.62 min, while the lowest concentration (9 × 10^−6^ M) caused a decrease of only an average of −15.9 ± 3.39% with a t_50_ equal to 1.32 ± 0.26 min. All observed changes were reversible, and heart rate returned to the baseline value with an RT_50_ time of 2.65 ± 0.93 min for verapamil at a concentration of 5 × 10^−5^ M. The determined IC_50_ value was equal to 1.685 × 10^−5^ M (Figure 2(B)). The *a* coefficient values also showed that the dynamics of the decrease in heart contraction frequency and recovery to the control range differed between concentrations. Interestingly, after finishing the perfusion of the heart with verapamil at the highest tested concentration, restoration of the basic frequency of heart contraction occurred with the highest dynamic (Table 1).

**Table 1.** The mean values (±SD) of *a* coefficient and percentage changes of heart contraction frequency determined for each tested concentration of verapamil.

A coefficient																								
Time. min		3	4	4.5	5	5.5	6	7	8	9	10	11	12	13	13.5	14	14.5	15	16	17	18	19	20	21
Saline	** **	0.1 ± 1.04	−0.2 ± 0.95	−1.0 ± 1.31	−0.2 ± 1.79	1.0 ± 1.71	0.0 ± 2.36	−0.5 ± 1.03	−0.1 ± 0.76	−0.5 ± 0.81	−0.3 ± 0.54	0.2 ± 0.73	−0.4 ± 0.52	0.2 ± 0.67	−0.1 ± 2.13	0.0 ± 2.72	−0.1 ± 2.01	1.0 ± 1.86	0.7 ± 1.24	−0.1 ± 0.64	0.0 ± 0.69	0.3 ± 0.70	0.2 ± 0.77	0.0 ± 0.39
ver 9 × 10^−6^ M	** **	−0.3 ± 0.45	−0.2 ± 1.30	−10.8 ± 7.32	−4.1 ± 0.77	−2.5 ± 0.27	−3.0 ± 0.05	−1.4 ± 0.06	−1.3 ± 0.27	−0.5 ± 0.16	−0.5 ± 0.50	−0.9 ± 0.25	−0.5 ± 0.85	−0.2 ± 0.94	3.1 ± 2.25	5.7 ± 1.82	5.6 ± 0.42	5.7 ± 1.44	3.7 ± 1.12	0.8 ± 1.07	1.0 ± 0.87	−0.2 ± 0.70	0.7 ± 1.19	−0.2 ± 1.19
** **	** **	ns	ns	ns	ns	ns	ns	ns	ns	ns	ns	ns	ns	ns	ns	ns	ns	ns	ns	ns	ns	ns	ns	ns
ver 1 × 10^−5^ M	** **	2.03	−1.9 ± 3.42	−12.6 ± 9.76	−8.8 ± 8.20	−10.2 ± 8.82	−5.0 ± 3.63	−3.4 ± 3.09	−1.5 ± 1.07	−1.0 ± 1.68	−0.8 ± 1.25	−0.1 ± 1.29	−0.5 ± 0.77	−0.7 ± 1.75	3.6 ± 6.19	13.4 ± 12.38	15.1 ± 10.22	7.1 ± 5.52	1.8 ± 3.34	−0.2 ± 2.92	0.4 ± 3.23	0.9 ± 3.00	1.2 ± 4.30	0.6 ± 3.54
	** **	ns	ns	*	ns	*	ns	ns	ns	ns	ns	ns	ns	ns	ns	**	***	ns	ns	ns	ns	ns	ns	ns
ver 2 × 10−^5^ M	** **	−0.3 ± 2.00	−1.2 ± 1.61	−21.0 ± 16.82	−34.1 ± 13.01	−13.3 ± 8.74	−5.1 ± 4.51	−3.2 ± 2.79	−3.3 ± 3.19	−2.4 ± 2.45	−2.8 ± 4.32	−1.1 ± 3.68	0.1 ± 1.65	0.3 ± 1.58	5.4 ± 11.61	9.2 ± 5.72	24.8 ± 14.07	7.7 ± 6.10	5.1 ± 3.63	2.0 ± 2.55	2.5 ± 2.99	2.7 ± 3.01	2.2 ± 3.54	2.0 ± 2.44
** **	** **	ns	ns	****	****	**	ns	ns	ns	ns	ns	ns	ns	ns	ns	ns	****	ns	ns	ns	ns	ns	ns	ns
ver 3 × 10^−5^ M	** **	−0.8 ± 0.53	0.7 ± 2.25	−29.6 ± 8.95	−46.6 ± 10.91	−21.9 ± 6.76	−22.0 ± 5.80	−5.5 ± 2.46	−4.8 ± 1.50	−2.3 ± 1.54	−1.3 ± 2.33	−1.4 ± 3.54	−2.0 ± 3.13	−0.3 ± 1.16	6.5 ± 4.62	6.7 ± 3.29	13.5 ± 6.46	14.3 ± 7.21	7.8 ± 2.34	7.7 ± 4.01	5.3 ± 2.86	4.6 ± 1.98	3.0 ± 2.17	2.4 ± 0.89
** **	** **	ns	ns	****	****	****	****	ns	ns	ns	ns	ns	ns	ns	ns	ns	**	**	ns	ns	ns	ns	ns	ns
ver 5 × 10^−5^ M	** **	0.4 ± 2.40	−0.6 ± 1.08	−50.2 ± 27.80	−49.1 ± 33.18	−54.0 ± 50.77	−23.7 ± 41.63	−6.1 ± 11.01	−3.7 ± 10.59	0.0 ± 0.13	0.1 ± 0.52	0.0 ± 0.08	0.1 ± 0.23	1.0 ± 3.69	−0.5 ± 1.91	6.8 ± 15.76	8.8 ± 22.85	32.6 ± 49.24	30.7 ± 31.28	11.3 ± 14.07	14.8 ± 22.66	4.6 ± 6.44	4.0 ± 6.24	−0.8 ± 5.14
** **	** **	ns	ns	****	****	****	****	ns	ns	ns	ns	ns	ns	ns	ns	ns	ns	****	****	*	***	ns	ns	ns

Percentage change																								
Time. min	2	3	4	4.5	5	5.5	6	7	8	9	10	11	12	13	13.5	14	14.5	15	16	17	18	19	20	21
Saline	−0.4 ± 0.85	−0.3 ± 1.12	−0.5 ± 1.06	−1.0 ± 0.85	−1.0 ± 0.85	−0.5 ± 1.42	−0.5 ± 1.51	−1.0 ± 1.67	−1.2 ± 1.85	−1.6 ± 1.99	−1.9 ± 1.83	−1.7 ± 2.07	−2.2 ± 2.30	−2.0 ± 2.49	−2.0 ± 2.41	−2.0 ± 1.70	−2.0 ± 1.42	−1.5 ± 1.04	−0.9 ± 1.86	−1.0 ± 2.11	−1.0 ± 2.34	−0.8 ± 2.08	−0.6 ± 2.42	−0.6 ± 2.18
ver 9 × 10^−6^ M	0.3 ± 0.96	−0.1 ± 1.02	−0.3 ± 1.60	−5.7 ± 3.89	−7.7 ± 3.82	−9.0 ± 3.83	−10.5 ± 3.84	−11.9 ± 3.83	−13.2 ± 3.79	−13.7 ± 3.78	−14.2 ± 3.85	−15.2 ± 3.84	−15.7 ± 3.73	−15.9 ± 3.39	−14.4 ± 3.48	−11.5 ± 3.88	−8.7 ± 3.86	−5.9 ± 4.03	−2.2 ± 3.73	−1.4 ± 3.79	−0.4 ± 3.75	−0.7 ± 3.85	0.0 ± 4.10	−0.2 ± 3.81
** **	ns	ns	ns	ns	ns	ns	*	**	**	**	***	***	***	****	***	*	ns	ns	ns	ns	ns	ns	ns	ns
ver 1 × 10−^5^ M	−0.6 ± 1.86	−0.5 ± 2.88	−2.4 ± 3.83	−8.6 ± 5.95	−13.1 ± 7.15	−18.2 ± 7.99	−20.7 ± 8.12	−24.1 ± 8.43	−25.6 ± 8.68	−26.6 ± 8.24	−27.4 ± 7.99	−27.5 ± 7.51	−28.0 ± 7.92	−28.7 ± 7.98	−26.9 ± 10.00	−20.2 ± 10.29	−12.7 ± 11.60	−9.1 ± 11.33	−7.3 ± 10.80	−7.5 ± 9.75	−7.1 ± 10.68	−6.2 ± 10.37	−5.0 ± 7.56	−4.4 ± 6.59
	ns	ns	ns	ns	**	****	****	****	****	****	****	****	****	****	****	****	**	ns	ns	ns	ns	ns	ns	ns
ver 2 × 10−^5^ M	−0.2 ± 1.67	−0.4 ± 2.87	−1.6 ± 2.59	−12.1 ± 8.14	−29.1 ± 9.43	−35.8 ± 8.73	−38.3 ± 8.74	−41.5 ± 7.18	−44.8 ± 7.59	−47.3 ± 8.31	−50.1 ± 6.96	−51.2 ± 7.05	−51.1 ± 6.81	−50.8 ± 7.88	−48.1 ± 11.37	−43.4 ± 11.59	−31.1 ± 11.71	−27.2 ± 10.67	−22.1 ± 12.62	−20.1 ± 11.88	−17.5 ± 10.89	−14.8 ± 10.81	−12.6 ± 9.71	−10.6 ± 9.65
** **	ns	ns	ns	**	****	****	****	****	****	****	****	****	****	****	****	****	****	****	****	****	****	****	**	*
ver 3 × 10^−5^ M	0.0 ± 1.49	−0.9 ± 1.02	−0.2 ± 3.24	−14.9 ± 1.88	−38.3 ± 4.39	−49.2 ± 3.59	−60.2 ± 3.44	−65.8 ± 5.15	− 70.6 ± 5.63	−72.9 ± 4.44	−74.2 ± 6.31	−75.7 ± 8.53	−77.6 ± 6.26	−77.9 ± 6.65	−74.6 ± 7.26	−71.3 ± 8.10	−64.6 ± 8.12	−57.4 ± 5.83	−49.6 ± 5.92	−42.0 ± 4.56	−36.7 ± 4.61	−32.1 ± 4.56	−29.1 ± 5.24	−26.7 ± 5.13
** **	ns	ns	ns	****	****	****	****	****	****	****	****	****	****	****	****	****	****	****	****	****	****	****	****	****
ver 5 × 10^−5^ M	−0.6 ± 2.92	−0.2 ± 3.30	−0.8 ± 3.43	−25.9 ± 13.14	−50.5 ± 26.60	−77.4 ± 27.48	−89.3 ± 17.09	−95.4 ± 10.70	−99.1 ± 3.41	−99.2 ± 3.28	−99.0 ± 3.80	−99.0 ± 3.72	−99.0 ± 3.95	−98.0 ± 7.64	−98.3 ± 6.69	−94.9 ± 13.86	−90.5 ± 18.07	−74.2 ± 28.52	−43.4 ± 28.90	−32.1 ± 25.16	−17.3 ± 12.74	−12.7 ± 10.92	−8.6 ± 10.91	−9.4 ± 10.14
** **	ns	ns	ns	****	****	****	****	****	****	****	****	****	****	****	****	****	****	****	****	****	****	**	*	*

Asterisks indicate statistically significant differences if *p*≤ 0.0001 (****), *p*≤ 0.001 (***), *p*≤ 0.01 (**) and *p*≤ 0.05 (*), or ns: statistically insignificant. *n*= 15; two-way ANOVA analysis with Dunn’s multi-comparison test. The table shows statistical differences in comparison to control (heart perfused with saline). A grey background corresponds to heart perfusion with verapamil solutions, white to perfusion with saline.

### Effects of α-solanine and α-chaconine on heart contractility

**Figure 3.**
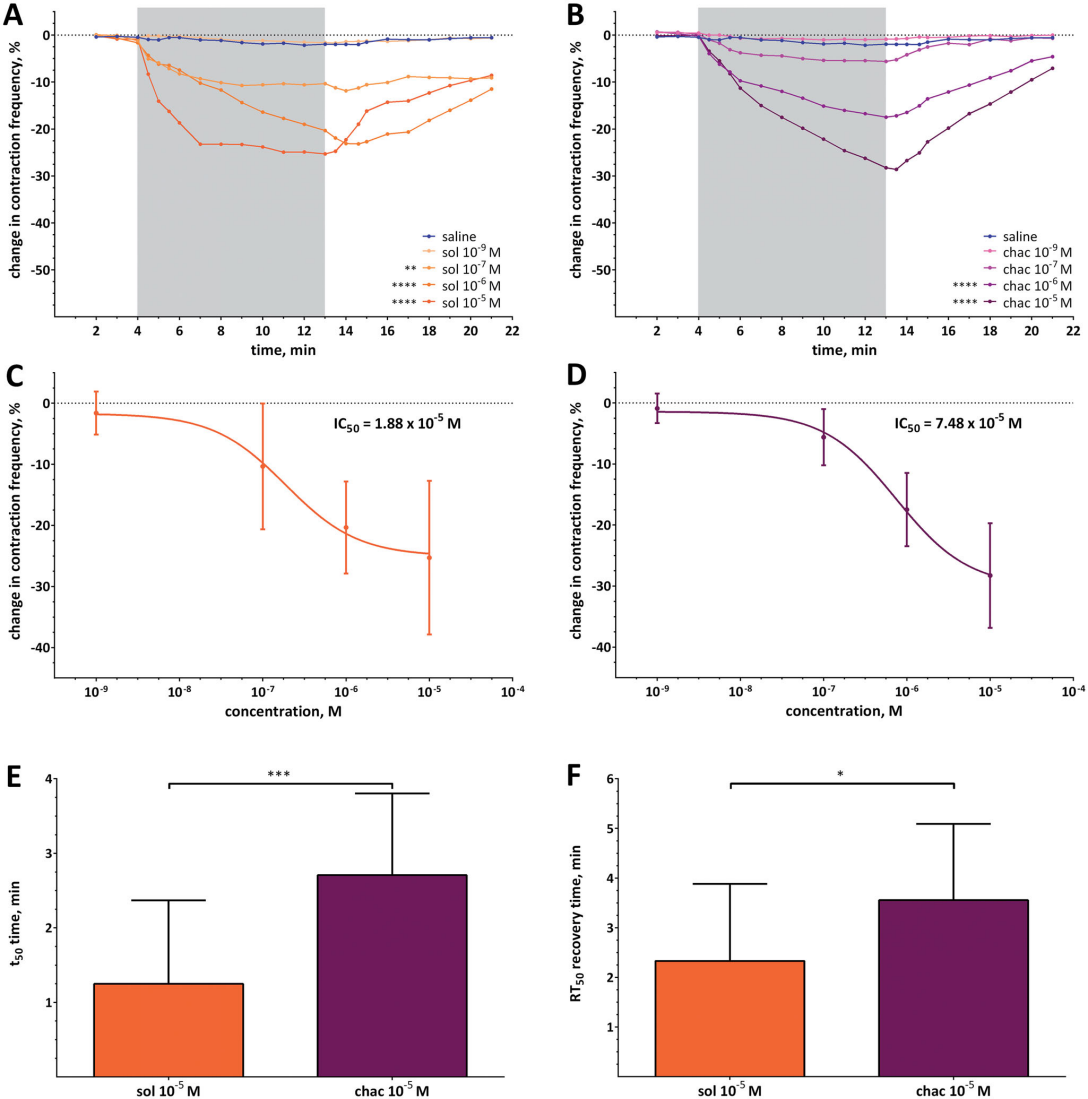
(A and B) Changes in the contraction frequency of the semi-isolated heart perfused with a solution of α-solanine (sol) or α-chaconine (chac) at different concentrations. Asterisks indicate statistically significant differences in comparison to control (saline) if *p*≤ 0.0001 (****) and *p*≤ 0.01 ***); *n*= 15; two-way ANOVA. The SD values are not shown to keep the clarity of diagrams, and the grey and white background indicates when the hearts were perfused with solutions of SGAs or saline, respectively. (C and D) dose–response curves for both tested glycoalkaloids at the last min of perfusion with tested SGAs. (E) T_50_ and (F) RT_50_ values for α-solanine and α-chaconine at a concentration of 10^−5^ M. Asterisks indicate statistically significant differences if *p*≤ 0.001 (****) and *p*≤ 0.05 (*); mean value ± SD; *n*= 15; Mann–Whitney test.

Both glycoalkaloids, similar to verapamil, caused reversible cardioinhibitory effects in a semi-isolated heart of *T. molitor* (Figure 3). The intensity of the observed changes in heart contraction frequency at the highest concentration tested (10^−5^ M) for both glycoalkaloids was similar to that caused by verapamil at a concentration of 10^−5^ M. The average maximal decrease in heart contraction frequency was −25.3 ± 12.6% and −28.3 ± 8.6% for α-solanine and α-chaconine (10^−5^ M), respectively, while the lowest concentration tested for both glycoalkaloids (10^−9^ M) did not change the frequency of heart contraction (Figure 3(A,B), Table 4). The determined IC_50_ concentrations were equal to IC_50_ = 1.88 × 10^−7^ M for α-solanine and IC_50_=7.48 × 10^−7^ M for α-chaconine (Figure 3(C,D)). Furthermore, the dynamics of the changes differed between α-solanine and α-chaconine. At a concentration of 10^−5^ M, the pace of decrease in heart contraction frequency was significantly higher for α-solanine than for α-chaconine. These results were confirmed by the values of the *a* coefficient (Table 2) and t_50_ parameters (Figure 3(E)). The *a* coefficient values for α-solanine were significantly lower (more negative values) than for α-chaconine between the 4.5th and 7th min. Therefore, the maximum decrease in the frequency of heart contraction caused by α-solanine was achieved in a shorter time than in the case of α-chaconine. The t_50_ for α-chaconine was longer more than twice that for α-solanine at 10^−5^ M (Mann–Whitney test, *p*= 0.0009). Furthermore, when comparing the effects at the highest tested concentration of SGAs, in the case of α-solanine after achieving the maximal effect, the further application of α-solanine did not change the frequency of heart contraction. However, in the case of α-chaconine, the intensity of the decrease in heart contraction frequency increased throughout the entire duration of glycoalkaloid application. Moreover, we also observed differences in the recovery time. The RT_50_ for α-chaconine was almost 1.5 times longer than that for α-solanine (Mann–Whitney test, *p* = 0.0294) (Figure 3(F)), which corresponds to the value of the *a* coefficient. For α-solanine, in the 15^th^ min of recording, the RT_50_ was significantly lower than for α-chaconine (Table 2).

**Table 2.** The mean values (±SD) of *a* coefficient and percentage changes of heart contraction frequency determined for each tested concentration of solanine and chaconine.

A coefficient																								
Time. min		3	4	4.5	5	5.5	6	7	8	9	10	11	12	13	13.5	14	14.5	15	16	17	18	19	20	21
Aaline		1.04	−0.2 ± 0.95	−1.0 ± 1.31	−0.2 ± 1.79	1.0 ± 1.71	0.0 ± 2.36	−0.5 ± 1.03	−0.1 ± 0.76	−0.5 ± 0.81	−0.3 ± 0.54	0.2 ± 0.73	−0.4 ± 0.52	0.67	−0.1 ± 2.13	0.0 ± 2.72	−0.1 ± 2.01	1.0 ± 1.86	0.7 ± 1.24	−0.1 ± 0.64	0.0 ± 0.69	0.3 ± 0.70	0.2 ± 0.77	0.0 ± 0.39
sol 10^−9^ M		0.0 ± 0.24	−0.2 ± 0.53	0.7 ± 1.84	−0.1 ± 2.81	−0.2 ± 1.40	0.1 ± 1.36	−0.3 ± 0.52	−0.3 ± 0.73	−0.4 ± 0.68	0.1 ± 0.79	−0.2 ± 0.42	−0.2 ± 0.42	−0.1 ± 0.58	−0.2 ± 0.72	0.6 ± 2.17	0.1 ± 2.29	0.2 ± 1.31	−0.1 ± 0.75	0.1 ± 0.40	0.2 ± 0.63	0.4 ± 0.77	−0.2 ± 0.86	0.1 ± 0.36
		ns	ns	ns	ns	ns	ns	ns	ns	ns	ns	ns	ns	ns	ns	ns	ns	ns	ns	ns	ns	ns	ns	ns
sol 10^−7^ M		−0.2 ± 0.94	−1.1 ± 2.50	−7.7 ± 8.05	−1.7 ± 4.69	−2.4 ± 6.36	−2.3 ± 2.39	−1.0 ± 3.81	−0.9 ± 2.56	−0.6 ± 2.24	0.1 ± 2.67	0.2 ± 1.76	−0.2 ± 2.08	0.2 ± 1.67	−1.7 ± 2.97	−1.3 ± 3.19	1.2 ± 2.39	1.4 ± 2.69	0.5 ± 2.59	1.3 ± 1.51	−0.2 ± 1.55	−0.1 ± 2.40	−0.2 ± 1.77	0.2 ± 2.12
		ns	ns	****	ns	*	ns	ns	ns	ns	ns	ns	ns	ns	ns	ns	ns	ns	ns	ns	ns	ns	ns	ns
sol 10^−6^ M		−0.5 ± 0.98	−1.0 ± 2.20	−5.4 ± 3.66	−3.7 ± 3.86	−0.5 ± 4.07	−2.0 ± 4.13	−2.8 ± 2.73	−1.5 ± 3.00	−2.7 ± 3.71	−2.0 ± 2.51	−1.4 ± 2.19	−1.3 ± 1.97	−1.3 ± 2.79	−3.1 ± 6.43	−2.4 ± 4.16	−0.2 ± 3.96	1.0 ± 2.61	1.6 ± 2.08	0.4 ± 2.14	2.5 ± 1.56	2.1 ± 1.57	2.2 ± 2.07	2.4 ± 3.25
		ns	ns	***	*	ns	ns	ns	ns	ns	ns	ns	ns	ns	ns	ns	ns	ns	ns	ns	ns	ns	ns	ns
sol 10^−5^ M		−0.7 ± 2.06	0.0 ± 1.82	−14.9 ± 9.83	−11.5 ± 8.01	−4.4 ± 6.65	−4.9 ± 9.01	−4.5 ± 9.06	0.0 ± 2.96	−0.1 ± 2.15	−0.5 ± 2.20	−1.1 ± 2.22	0.1 ± 2.30	−0.4 ± 2.11	1.1 ± 4.38	4.9 ± 6.46	5.9 ± 8.98	6.4 ± 2.60	1.9 ± 3.81	0.3 ± 2.54	1.7 ± 3.00	1.6 ± 2.87	1.1 ± 2.76	1.1 ± 2.68
		ns	ns	****	****	****	****	**	ns	ns	ns	ns	ns	ns	ns	****	****	****	ns	ns	ns	ns	ns	ns
chac 10^−9^ M		−0.1 ± 0.25	0.0 ± 0.56	−0.9 ± 1.35	0.0 ± 1.35	−0.6 ± 2.35	−0.8 ± 1.26	−0.1 ± 0.47	0.1 ± 0.78	−0.2 ± 0.75	−0.1 ± 0.93	0.1 ± 0.48	−0.1 ± 0.77	0.1 ± 0.81	0.1 ± 0.52	0.2 ± 0.96	0.6 ± 1.56	−0.2 ± 1.29	0.0 ± 0.51	0.3 ± 0.73	0.1 ± 0.56	−0.2 ± 0.68	0.2 ± 0.60	0.1 ± 0.98
		ns	ns	ns	ns	ns	ns	ns	ns	ns	ns	ns	ns	ns	ns	ns	ns	ns	ns	ns	ns	ns	ns	ns
chac 10^−7^ M		−0.1 ± 0.78	−0.4 ± 0.81	−2.4 ± 3.90	−1.5 ± 3.55	−2.8 ± 2.31	−1.3 ± 1.56	−0.5 ± 1.04	−0.2 ± 0.99	−0.6 ± 1.02	−0.4 ± 0.66	−0.1 ± 0.53	0.0 ± 0.55	−0.2 ± 0.71	0.7 ± 2.51	2.1 ± 2.64	2.1 ± 2.68	1.2 ± 2.05	0.8 ± 1.36	−0.3 ± 1.14	1.2 ± 1.92	−0.4 ± 1.59	0.6 ± 1.30	−0.1 ± 1.33
		ns	ns	ns	ns	**	ns	ns	ns	ns	ns	ns	ns	ns	ns	ns	ns	ns	ns	ns	ns	ns	ns	ns
chac 10^−6^ M		−0.4 ± 0.54	−0.5 ± 0.76	−7.5 ± 3.64	−4.5 ± 2.74	−3.3 ± 2.80	−3.7 ± 2.86	−1.1 ± 1.33	−1.1 ± 0.97	−1.5 ± 1.04	−1.7 ± 1.29	−0.9 ± 0.83	−0.7 ± 0.82	−0.7 ± 0.60	0.5 ± 2.86	1.4 ± 2.44	2.8 ± 2.56	3.0 ± 3.23	1.5 ± 1.21	1.5 ± 1.56	1.5 ± 1.24	1.5 ± 1.79	2.1 ± 2.27	0.9 ± 1.84
		ns	ns	****	***	***	**	ns	ns	ns	ns	ns	ns	ns	ns	ns	ns	ns	ns	ns	ns	ns	ns	ns
chac 10^−5^ M		0.0 ± 0.70	0.1 ± 1.22	−6.8 ± 5.01	−4.1 ± 3.75	−5.5 ± 5.39	−6.2 ± 4.48	−3.7 ± 2.62	−2.5 ± 1.84	−2.3 ± 1.76	−2.3 ± 1.34	−2.4 ± 1.35	−1.6 ± 2.44	−2.0 ± 1.62	−0.7 ± 4.08	3.7 ± 4.15	3.3 ± 3.30	4.6 ± 2.74	2.9 ± 2.12	3.1 ± 2.56	2.1 ± 1.54	2.5 ± 2.31	2.6 ± 1.87	2.4 ± 2.59
		ns	ns	****	**	****	****	*	ns	ns	ns	ns	ns	ns	ns	**	*	**	ns	ns	ns	ns	ns	ns
sol 10^−5^ M *vs.*		ns	ns	****	****	ns	ns	ns	ns	ns	ns	ns	ns	ns	ns	ns	ns	ns	ns	ns	ns	ns	ns	ns
chac 10^−5^ M																								

Percentage change																								
Time. min	2	3	4	4.5	5	5.5	6	7	8	9	10	11	12	13	13.5	14	14.5	15	16	17	18	19	20	21
Saline	−0.4 ± 0.85	−0.3 ± 1.12	−0.5 ± 1.06	−1.0 ± 0.85	−1.0 ± 0.85	−0.5 ± 1.42	−0.5 ± 1.51	−1.0 ± 1.67	−1.2 ± 1.85	−1.6 ± 1.99	−1.9 ± 1.83	−1.7 ± 2.07	−2.2 ± 2.30	−2.0 ± 2.49	−2.0 ± 2.41	−2.0 ± 1.70	−2.0 ± 1.42	−1.5 ± 1.04	−0.9 ± 1.86	−1.0 ± 2.11	−1.0 ± 2.34	−0.8 ± 2.08	−0.6 ± 2.42	−0.6 ± 2.18
sol 10^−9^ M	−0.3 ± 1.76	−0.3 ± 1.77	−0.5 ± 1.77	−0.2 ± 1.88	−0.2 ± 2.09	−0.3 ± 2.23	−0.3 ± 2.36	−0.6 ± 2.67	−0.9 ± 2.85	−1.3 ± 3.14	−1.2 ± 3.43	−1.3 ± 3.47	−1.5 ± 3.32	−1.6 ± 3.53	−1.7 ± 3.48	−1.4 ± 3.75	−1.3 ± 3.41	−1.3 ± 3.11	−1.3 ± 2.69	−1.2 ± 2.73	−1.0 ± 2.51	−0.6 ± 2.62	−0.8 ± 2.45	−0.6 ± 2.33
	ns	ns	ns	ns	ns	ns	ns	ns	ns	ns	ns	ns	ns	ns	ns	ns	ns	ns	ns	ns	ns	ns	ns	ns
sol 10^−7^ M	0.1 ± 0.66	−0.1 ± 0.97	−1.2 ± 2.47	−5.1 ± 4.05	−5.9 ± 4.71	−7.1 ± 6.13	−8.3 ± 6.28	−9.3 ± 6.92	−10.2 ± 7.90	−10.8 ± 8.58	−10.6 ± 9.29	−10.4 ± 9.81	−10.6 ± 10.08	−10.4 ± 10.31	−11.2 ± 10.45	−11.9 ± 10.70	−11.3 ± 11.07	−10.6 ± 11.46	−10.1 ± 11.83	−8.8 ± 11.94	−9.0 ± 11.84	−9.1 ± 11.26	−9.3 ± 1 0.85	−9.1 ± 9.73
	ns	ns	ns	ns	ns	ns	*	**	**	**	**	**	**	**	**	***	**	**	**	*	*	**	**	**
sol 10^−6^ M	−0.1 ± 0.88	−0.7 ± 0.86	−1.7 ± 2.35	−4.3 ± 3.26	−6.2 ± 3.29	−6.4 ± 3.24	−7.5 ± 3.93	−10.3 ± 5.33	−11.7 ± 6.63	−14.4 ± 6.15	−16.4 ± 5.48	−17.8 ± 5.53	−19.0 ± 6.12	−20.4 ± 7.53	−21.9 ± 7.63	−23.1 ± 6.90	−23.2 ± 7.05	−22.7 ± 7.77	−21.1 ± 7.61	−20.7 ± 7.82	−18.2 ± 7.49	−16.0 ± 6.73	−13.9 ± 6.84	−11.5 ± 5.84
	ns	ns	ns	ns	ns	ns	*	**	***	****	****	****	****	****	****	****	****	****	****	****	****	****	****	***
sol 10^−5^ M	−0.2 ± 0.82	−0.8 ± 2.40	−0.9 ± 2.11	−8.3 ± 4.78	−14.1 ± 6.32	−16.3 ± 7.42	−18.7 ± 7.91	−23.3 ± 12.49	−23.2 ± 11.72	−23.3 ± 10.95	−23.8 ± 11.51	−24.9 ± 11.56	−24.9 ± 12.16	−25.3 ± 12.58	−24.8 ± 12.35	−22.3 ± 13.10	−19.0 ± 13.09	−16.2 ± 12.60	−14.3 ± 13.26	−14.0 ± 13.01	−12.3 ± 12.50	−10.8 ± 11.36	−9.7 ± 10.47	−8.6 ± 9.36
	ns	ns	ns	*	****	****	****	****	****	****	****	****	****	****	****	****	****	****	****	****	****	***	**	*
chac 10^−9^ M	0.6 ± 1.72	0.4 ± 1.66	0.4 ± 1.63	0.0 ± 1.66	0.0 ± 1.53	−0.3 ± 2.01	−0.7 ± 1.84	−0.8 ± 2.05	−0.7 ± 2.22	−0.9 ± 2.25	−1.0 ± 2.16	−0.9 ± 2.23	−1.0 ± 2.25	−0.9 ± 2.42	−0.8 ± 2.46	−0.7 ± 2.24	−0.4 ± 2.31	−0.5 ± 2.00	−0.5 ± 2.10	−0.2 ± 1.89	−0.1 ± 2.00	−0.3 ± 1.96	−0.1 ± 1.82	0.0 ± 1.72
	ns	ns	ns	ns	ns	ns	ns	ns	ns	ns	ns	ns	ns	ns	ns	ns	ns	ns	ns	ns	ns	ns	ns	ns
chac 10^−7^ M	0.6 ± 1.58	0.6 ± 1.23	0.2 ± 1.08	−1.0 ± 2.11	−1.7 ± 2.56	−3.1 ± 2.96	−3.8 ± 3.52	−4.3 ± 3.92	−4.5 ± 4.31	−5.0 ± 4.44	−5.4 ± 4.21	−5.5 ± 4.08	−5.4 ± 4.17	−5.6 ± 4.60	−5.2 ± 4.07	−4.2 ± 3.92	−3.1 ± 3.45	−2.6 ± 3.18	−1.7 ± 2.55	−2.0 ± 2.46	−0.8 ± 2.34	−1.2 ± 2.19	−0.6 ± 1.93	−0.7 ± 1.93
	ns	ns	ns	ns	ns	ns	ns	ns	ns	ns	ns	ns	ns	ns	ns	ns	ns	ns	ns	ns	ns	ns	ns	ns
chac 10^−6^ M	0.6 ± 0.88	0.3 ± 0.91	−0.2 ± 1.30	−4.0 ± 1.94	−6.2 ± 2.34	−7.9 ± 3.02	−9.7 ± 3.65	−10.9 ± 3.81	−12.0 ± 4.20	−13.5 ± 4.95	−15.1 ± 5.77	−16.1 ± 5.61	−16.8 ± 5.81	−17.5 ± 6.00	−17.2 ± 5.38	−16.5 ± 4.99	−15.1 ± 4.63	−13.6 ± 4.24	−12.1 ± 4.20	−10.7 ± 4.05	−9.1 ± 4.00	−7.6 ± 4.25	−5.5 ± 3.97	−4.6 ± 3.51
	ns	ns	ns	ns	**	****	****	****	****	****	****	****	****	****	****	****	****	****	****	****	****	****	*	ns
chac 10^−5^ M	−0.1 ± 0.79	−0.1 ± 0.81	0.0 ± 1.10	−3.4 ± 2.11	−5.5 ± 2.11	−8.2 ± 3.79	−11.3 ± 5.39	−15.0 ± 6.87	−17.5 ± 7.72	−19.8 ± 8.36	−22.2 ± 8.09	−24.6 ± 8.07	−26.2 ± 8.60	−28.3 ± 8.57	−28.6 ± 8.39	−26.7 ± 9.06	−25.1 ± 9.49	−22.8 ± 9.73	−19.8 ± 9.89	−16.7 ± 8.58	−14.7 ± 8.63	−12.2 ± 8.05	−9.5 ± 6.93	−7.1 ± 5.70
	ns	ns	ns	ns	*	****	****	****	****	****	****	****	****	****	****	****	****	****	****	****	****	****	****	***
sol 10^−5^ M vs	ns	ns	ns	**	**	**	*	**	ns	ns	ns	ns	ns	ns	ns	ns	ns	*	ns	ns	ns	ns	ns	ns
chac 10^−5^ M																								

Asterisks indicate statistically significant differences if *p* ≤ 0.0001 (****), *p*≤ 0.001 (***), *p*≤ 0.01 (**), *p*≤ 0.05 (*), or ns – statistically insignificant. *n*= 15; two-way ANOVA analysis with Dunn’s multicomparison test. The table shows statistical differences in comparison to control (heart perfused with saline) and additionaly a comparison between solanine and chaconine in concentration 10−^5^ M. A blue background corresponds to heart perfusion with glycoalkaloid solutions, white to perfusion with saline.

### Effects of the interaction of verapamil and glycoalkaloids on heart contractility

#### Continuous perfusion

**Figure 4.**
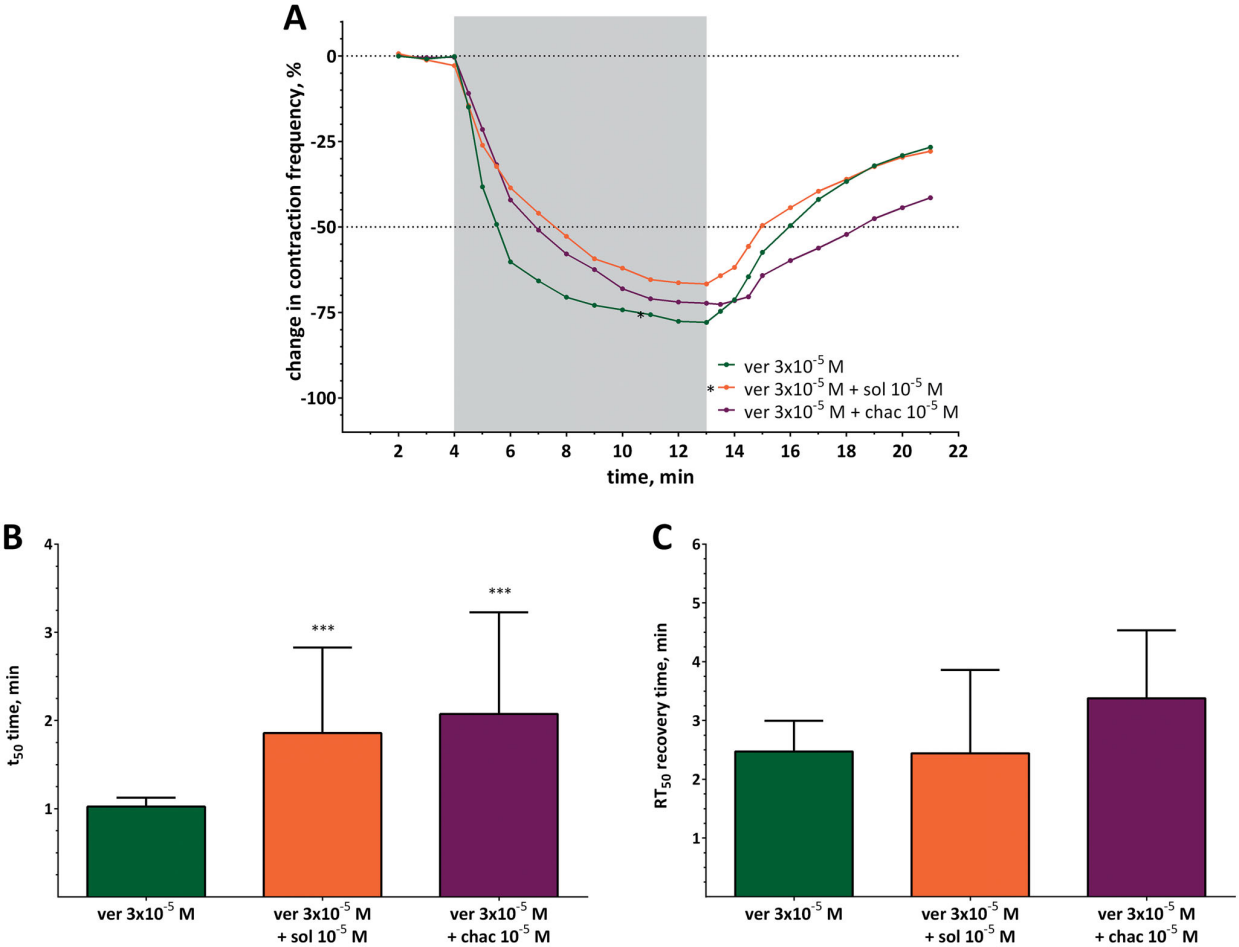
(A) Changes in contraction frequency of semi-isolated heart treated with verapamil (ver) or with mixtures of verapamil and glycoalkaloids (sol, chac). The SD values are not shown to keep the clarity of the diagram, and the grey and white background indicates when the hearts were perfused with solutions of tested compounds or saline, respectively. (B) T_50_ and C) RT_50_ values for verapamil and mixes of verapamil and glycoalkaloids. Asterisks indicate statistically significant differences in comparison to verapamil if *p*≤ 0.05 (*), *p*≤ 0.001 (***); *n*= 15; two-way ANOVA (A) and Kruskal–Wallis test (B and C).

Perfusion of a semi-isolated heart with a solution of verapamil at concentration of 3 × 10^−5^ M caused an average decrease in heart contraction frequency of approximately −77.9 ± 6.65%. When verapamil was applied simultaneously with one of the glycoalkaloids, the observed effects differed from those observed for hearts perfused only with verapamil (Figure 4(A)). The effects were especially noticeable for a mixture of verapamil and α-solanine. When this mixture was used, the decrease in heart contraction frequency was significantly lower than that observed for verapamil (*p* ≤ 0.01; two-way ANOVA; *q* = 4.173; DF = 42) and reached the maximum value equal to −77.9 ± 6.65% and −66.7 ± 14.19% for verapamil and the mixture of ver + sol, respectively (Table 3). It could be assumed that if both compounds cause cardioinhibition, an additive or synergistic effect would be observed. However, experiments showed that verapamil and α-solanine act antagonistically when applied together, as indicated by the TI_mix_ and TU_mix_ values (Table 1). The antagonistic effect of α-solanine on verapamil activity was also confirmed by the *a* coefficient and t_50_. The value of the *a* coefficient was significantly higher between the 4.5th and 6th min of registration (Table 3) which indicates that the pace of decrease in heart contraction frequency was less intense for the mixture of ver + sol than for verapamil. Moreover, the t_50_ for the ver + sol mixture (1.86 ± 0.97 min) was almost 1.8 times longer than for verapamil (1.02 ± 0.10 min) (Figure 4(B)). Interestingly, the *a* coefficient did not differ significantly between verapamil and the ver + sol mixture during recovery time. The same was observed for RT_50_ (Figure 4(C), Table 3).

α-Chaconine also acted antagonistically with respect to verapamil (Table 4) and weakened the effect evoked by verapamil, but its activity was lower (*p* > 0.05; two-way ANOVA; *q* = 0.452; DF = 42) than that of α-solanine, and the maximal decrease in heart contraction frequency for the ver + chac mixture was equal to −72.3 ± 11.43%. However, it caused an elongation of the t_50_ time from 1.02 ± 0.10 min for verapamil to 2.07 ± 1.15 min when applied simultaneously with verapamil and prolonged the RT_50_ time, but the changes were statistically insignificant. These changes were coupled with differentiation in the value of *a* coefficient at the 4.5th, 5th, 13.5th and 14.5th min of registration between verapamil and the mixture of ver + chac (Table 3).

**Table 3.** The mean values (±SD) of *a* coefficient and percentage changes of heart contraction frequency determined for mixes of verapamil and glycoalkaloids.

A coefficient																								
Time. min		3	4	4.5	5	5.5	6	7	8	9	10	11	12	13	13.5	14	14.5	15	16	17	18	19	20	21
**ver 3 × 10** ^−^ **^5^ M**	** **	−0.8 ± 0.53	0.7 ± 2.25	−29.6 ± 8.95	−46.6 ± 10.91	−21.9 ± 6.76	−22.0 ± 5.80	−5.5 ± 2.46	−4.8 ± 1.50	−2.3 ± 1.54	−1.3 ± 2.33	−1.4 ± 3.54	−2.0 ± 3.13	−0.3 ± 1.16	6.5 ± 4.62	6.7 ± 3.29	13.5 ± 6.46	14.3 ± 7.21	7.8 ± 2.34	7.7 ± 4.01	5.3 ± 2.86	4.6 ± 1.98	3.0 ± 2.17	2.4 ± 0.89
**ver 3 × 10** ^−^ **^5^ M +**	** **	−1.8 ± 2.72	−−1.7 ± 3.77	−23.3 ± 3.95	−23.3 ± 3.95	−12.5 ± 7.11	−12.5 ± 7.11	−7.5 ± 3.23	−6.7 ± 4.04	−6.6 ± 8.34	−2.8 ± 2.03	−3.3 ± 3.69	−0.9 ± 1.83	−0.4 ± 3.50	4.9 ± 6.67	4.9 ± 6.67	12.2 ± 9.31	12.2 ± 9.31	5.2 ± 5.37	4.8 ± 4.01	3.5 ± 5.18	3.7 ± 3.62	2.8 ± 3.30	1.7 ± 2.64
**sol 10** ^−^ **^5^ M**	** **	ns	ns	**	****	****	****	ns	ns	ns	ns	ns	ns	ns	ns	ns	ns	ns	ns	ns	ns	ns	ns	ns
**ver 3 × 10** ^−^ **^5^ M +**	** **	−0.6 ± 1.51	0.1 ± 1.71	−21.1 ± 10.96	−21.1 ± 10.96	−20.6 ± 9.77	−20.9 ± 9.08	−8.8 ± 4.45	−7.0 ± 4.85	−4.6 ± 2.72	−5.6 ± 8.31	−2.9 ± 3.09	−0.9 ± 2.65	−0.3 ± 0.57	−0.7 ± 1.13	2.2 ± 4.05	2.1 ± 4.12	12.5 ± 11.69	4.4 ± 2.82	3.7 ± 2.09	4.0 ± 2.46	4.6 ± 2.81	3.2 ± 2.36	2.9 ± 1.44
**chac 10** ^−^ **^5^ M**	** **	ns	ns	****	****	ns	ns	ns	ns	ns	ns	ns	ns	ns	****	*	****	ns	ns	ns	ns	ns	ns	ns

Percentage change																								
Time. min	2	3	4	4.5	5	5.5	6	7	8	9	10	11	12	13	13.5	14	14.5	15	16	17	18	19	20	21
ver 3 × 10^−5^ M	0.0 ± 1.49	−0.9 ± 1.02	−0.2 ± 3.24	−14.9 ± 1.88	−38.3 ± 4.39	−49.2 ± 3.59	−60.2 ± 3.44	−65.8 ± 5.15	−70.6 ± 5.63	−72.9 ± 4.44	−74.2 ± 6.31	−75.7 ± 8.53	−77.6 ± 6.26	−77.9 ± 6.65	−74.6 ± 7.26	−71.3 ± 8.10	−64.6 ± 8.12	−57.4 ± 5.83	−49.6 ± 5.92	−42.0 ± 4.56	−36.7 ± 4.61	−32.1 ± 4.56	−29.1 ± 5.24	−26.7 ± 5.13
ver 3 × 10^−5^ M +	0.7 ± 2.72	−1.2 ± 2.31	−2.8 ± 4.31	−14.5 ± 4.10	−26.1 ± 4.81	−32.3 ± 6.93	−38.6 ± 9.91	−46.0 ± 10.19	−52.8 ± 10.86	−59.3 ± 12.24	−62.1 ± 12.50	−−65.4 ± 13.86	−66.3 ± 14.01	−66.7 ± 14.19	−64.2 ± 14.75	−61.8 ± 15.99	−55.7 ± 14.69	−49.6 ± 14.80	−44.4 ± 14.44	−39.6 ± 14.04	−36.0 ± 14.02	−32.4 ± 13.94	−29.6 ± 14.67	−27.9 ± 15.06
sol 10^−5^ M	ns	ns	ns	**	****	****	****	****	****	***	**	*	**	**	*	*	*	ns	ns	ns	ns	ns	ns	ns
ver 3 × 10^−5^ M +	0.0 ± 1.67	−0.5 ± 2.39	−0.4 ± 3.04	−11.0 ± 5.44	−21.5 ± 10.46	−31.8 ± 13.22	−42.1 ± 16.95	−50.9 ± 17.52	−57.9 ± 15.64	−62.5 ± 14.06	−68.1 ± 12.35	−71.0 ± 11.98	−71.9 ± 11.47	−72.3 ± 11.43	−72.6 ± 11.42	−71.5 ± 11.65	−70.4 ± 12.22	−64.2 ± 11.00	−59.8 ± 10.58	−56.2 ± 10.72	−52.2 ± 10.09	−47.6 ± 9.44	−44.4 ± 9.41	−41.5 ± 9.58
chac 10^−5^ M	ns	ns	ns	****	****	***	***	**	**	*	ns	ns	ns	ns	ns	ns	ns	ns	*	***	****	****	***	***

Asterisks indicate statistically significant differences if *p*≤ 0.0001 (****), *p*≤ 0.001 (***), *p*≤ 0.01 (**), *p*≤ 0.05 (*), or ns – statistically insignificant. *n*= 15; two-way ANOVA analysis with Dunn’s multicomparison test. The table shows statistical differnces in comparison to verapamil in concentration 3 × 10^−5^ M. A blue background corresponds to heart perfusion with verapamil and glycoalkaloid mixes, white to perfusion with saline.

**Table 4.** The TI_max_ and TU_max_ values determined for heart contraction frequency in the 9th and 13th minute of recording.

Substances	TI_mix_	TU_mix_
Heart contraction frequency for mixes of verapamil and glycoalkaloids 13th min		
ver + sol	0.65 — antagonism	3.49 — antagonism
ver + chac	0.68 — antagonism	3.48 — antagonism
Heart contraction frequency for verapamil and glycoalkaloids with pulse application		
9 min		
ver + sol	0.49 — antagonism	1.96 — antagonism
ver + chac	0.49 — antagonism	1.95 — antagonism
13 min		
ver + sol	0.73 — antagonism	3.92 – antagonism
ver + chac	0.67 — antagonism	3.43 — antagonism

#### Pulse application

**Figure 5.**
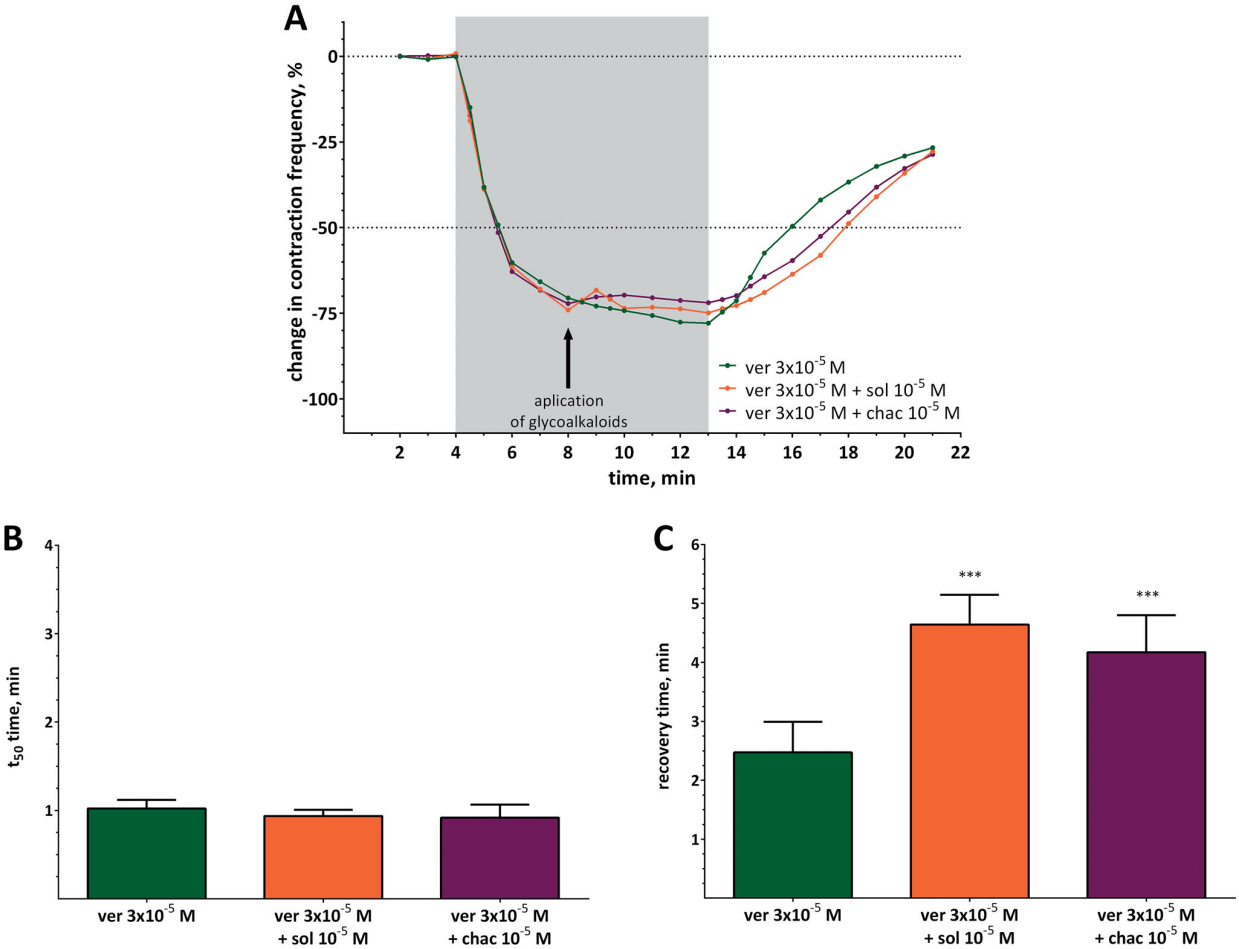
(A) Changes in contraction frequency of semi-isolated heart treated with verapamil (ver) and treated with glycoalkaloids (sol, chac) by pulse application. The SD values are not shown to keep the clarity of the diagram, and the grey and white background indicates when the hearts were perfused with solutions of verapamil or saline, respectively. The arrow indicates the time when the solutions of glycoalkaloids were applied. (B) T_50_ and C) RT_50_ values for verapamil and mixes of verapamil and glycoalkaloids. Asterisks indicate statistically significant differences in comparison to verapamil if *p*≤ 0.001 (***); *n*= 15–16; two-way ANOVA (A) and Kruskal–Wallis test (B and C).

The pulse application of glycoalkaloids during the perfusion of the semi-isolated heart with verapamil at a concentration of 3 × 10^−5^ M, as in the first experimental variant, also changed the cardioinhibitory effect evoked by verapamil (Figure 5(A)). We determined that the antagonistic effect was observed when the α-solanine or α-chaconine solutions had been applied to the verapamil-perfused heart, and the decrease in heart contraction frequency caused by verapamil was partially inhibited by both glycoalkaloids. Nevertheless, some differences between α-solanine and α-chaconine action could also be noticed. α-Solanine caused an increase in the *a* coefficient in the 8.5th and 9th min of recording of the heart activity registration Table 5). Thus, the decrease in heart contraction frequency was smaller than that in the case of verapamil. Nevertheless, at the next time points, the 9.5th and 10th min of registration, the values of *a* coefficient were even lower than that for verapamil, so the decrease in heart contraction frequency returned to a level similar to that for verapamil (Table 5). α-Chaconine increased the value of the *a* coefficient only in the 8.5th and 9th min of registration without changes at later time points. Therefore, the changes in heart contraction frequency evoked by ver + chac did not achieve the maximal cardioinhibitory effect expected for verapamil. Neither of the SGAs tested changed the t_50_ time (Figure 5(B)), but significantly prolonged the value of RT_50_ (Figure 5(C)). The resulting values were almost 1.9 and 1.7 times longer than that for verapamil.

**Table 5.** The mean values (±SD) of *a* coefficient and percentage changes of heart contraction frequency determined for heart perfused with verapamil in concentration 3 × 10^−5^ M and glycoalkaloids applied with pulsating application in 8 min.

A coefficient																								
Time. min		3	4	5	6	7	8	8.5	9	9.5	10	11	12	13	13.5	14	14.5	15	16	17	18	19	20	21
ver 3 × 10^−5^ M	** **	−0.8 ± 0.53	0.7 ± 2.25	−38.1 ± 1.56	−22.0 ± 1.45	−5.5 ± 2.46	−4.8 ± 1.50	−2.3 ± 1.54	−2.3 ± 1.54	−1.3 ± 2.33	−1.3 ± 2.33	−1.4 ± 3.54	−2.0 ± 3.13	−0.3 ± 1.16	6.5 ± 4.62	6.7 ± 3.29	13.5 ± 6.46	14.3 ± 7.21	7.8 ± 2.34	7.7 ± 4.01	5.3 ± 2.86	4.6 ± 1.98	3.0 ± 2.17	2.4 ± 0.89
ver 3 × 10^−5^ M +	** **	−0.6 ± 3.06	1.5 ± 3.52	−39.6 ± 1.86	−22.5 ± 1.99	−6.7 ± 1.29	−6.1 ± 1.79	5.5 ± 1.74	6.0 ± 1.30	−5.3 ± 1.06	−5.3 ± 1.06	0.3 ± 0.85	−0.4 ± 1.14	−1.2 ± 0.78	2.6 ± 1.96	1.7 ± 2.38	3.6 ± 1.55	4.0 ± 1.17	5.3 ± 1.16	5.6 ± 0.85	9.3 ± 3.70	7.8 ± 3.40	6.9 ± 3.30	6.3 ± 4.39
sol 10^−5^ M	** **	ns	ns	ns	ns	ns	ns	ns	****	****	***	ns	ns	ns	***	****	****	****	*	ns	***	**	***	***
ver 3 × 10^−5^ M +	** **	0.1 ± 2.35	0.1 ± 1.92	−38.5 ± 6.26	−23.9 ± 4.07	−6.1 ± 3.36	−4.0 ± 1.83	2.0 ± 1.53	2.1 ± 1.31	0.5 ± 1.24	0.5 ± 1.24	−0.8 ± 0.79	−0.8 ± 1.00	−0.6 ± 0.56	1.8 ± 1.72	2.3 ± 2.01	5.6 ± 3.41	5.5 ± 3.15	4.7 ± 1.91	7.1 ± 3.23	7.1 ± 3.50	7.3 ± 3.06	5.4 ± 2.01	4.1 ± 2.68
chac 10^−5^ M	** **	ns	ns	ns	ns	ns	ns	ns	****	****	ns	ns	ns	ns	****	****	****	****	**	ns	ns	*	*	ns

Percentage change																								
Time. min	2	3	4	5	6	7	8	8.5	9	9.5	10	11	12	13	13.5	14	14.5	15	16	17	18	19	20	21
ver 3 × 10^−5^ M	0.0 ± 1.49	−0.9 ± 1.02	−0.2 ± 3.24	−38.3 ± 4.39	−60.2 ± 3.44	−65.8 ± 5.15	−70.6 ± 5.63	−71.8 ± 5.02	−72.9 ± 4.44	−73.6 ± 5.33	−74.2 ± 6.31	−75.7 ± 8.53	−77.6 ± 6.26	−77.9 ± 6.65	−74.6 ± 7.26	−71.3 ± 8.10	−64.6 ± 8.12	−57.4 ± 5.83	−49.6 ± 5.92	−42.0 ± 4.56	−36.7 ± 4.61	−32.1 ± 4.56	−29.1 ± 5.24	−26.7 ± 5.13
ver 3 × 10^−5^ M +	−0.1 ± 2.74	−0.7 ± 3.13	0.8 ± 3.42	−38.9 ± 3.76	−61.3 ± 4.66	−68.0 ± 3.90	−74.0 ± 4.60	−71.3 ± 4.35	−68.3 ± 4.04	−70.9 ± 4.30	−73.6 ± 4.59	−73.3 ± 4.92	−73.7 ± 5.21	−74.9 ± 5.13	−73.6 ± 5.55	−72.8 ± 6.21	−71.0 ± 6.79	−69.0 ± 7.22	−63.6 ± 6.75	−58.1 ± 6.51	−48.8 ± 7.42	−41.0 ± 6.96	−34.1 ± 6.29	−27.8 ± 5.68
sol 10^−5^ M	ns	ns	ns	ns	ns	ns	ns	ns	ns	ns	ns	ns	ns	ns	ns	ns	*	****	****	****	****	***	ns	ns
ver 3 × 10^−5^ M +	0.1 ± 3.40	0.2 ± 2.70	0.3 ± 2.20	−38.2 ± 6.42	−62.1 ± 6.75	−68.2 ± 7.07	−72.2 ± 7.02	−71.2 ± 7.28	−70.2 ± 7.60	−70.0 ± 8.00	−69.7 ± 8.39	−70.5 ± 8.74	−71.3 ± 8.50	−71.9 ± 8.47	−71.0 ± 8.12	−69.9 ± 7.91	−67.1 ± 7.88	−64.3 ± 8.21	−59.6 ± 8.77	−52.5 ± 9.37	−45.5 ± 9.90	−38.2 ± 9.05	−32.8 ± 9.02	−28.6 ± 8.15
chac 10^−5^ M	ns	ns	ns	ns	ns	ns	ns	ns	ns	ns	ns	ns	*	*	ns	ns	ns	**	****	****	***	*	ns	ns

Asterisks indicate statistically significant differences if *p* ≤ 0.0001 (****), *p*≤ 0.001 (***), *p*≤ 0.01 (**), *p*≤ 0.05 (*), or ns – statistically insignificant. *n*= 15–16; two‐way ANOVA analysis with Dunn’s multicomparison test. The table shows statistical differences in comparison to verapamil in concentration 3 × 10^−5^ M. A blue background corresponds to heart perfusion with verapamil and glycoalkaloid mixes, white to perfusion with saline.

#### Distribution of the L-type calcium channel subunit α1 in tissues

**Figure 6.**
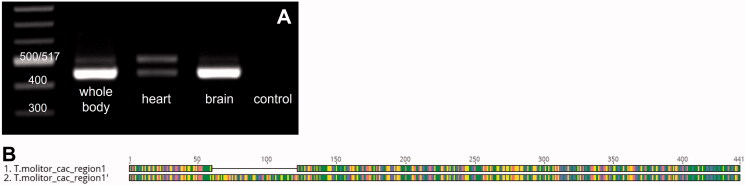
(A) Transcript distribution profile of L-type calcium channel in different tissues of *T. molitor* adults. The data represent samples of the whole body, heart and brain. Water was used as a control. (B) Graphical representation of two transcripts detected in tested tissues.

To confirm that the observed effect evoked in the heart is the result of inhibition of L-type calcium channels, we analysed the distribution of the transcript for the gene encoding the α1-subunit of the L-type calcium channel in different tissues of *T. molitor.* The experiments confirmed its expression in the heart and brain (Figure 6(A)). Moreover, we found two transcripts in the analysed regions *T. molitor*_cac_region1 and *T. molitor*_cac_region 1′ (Figure 6(B)), which differed by 60 bp in length. Although we performed only spatial distribution, the intensity of the bands (equal concentrations of template were used in RT-PCR and equal amount of mass marker and samples imposed on agarose gel) suggests that the 1′ form of a transcript dominated in the whole body and in the brain samples, while the amount of both transcripts was equal in the heart samples. No foreign or genomic DNA was found in the ‘no template control’ or ‘no RT control’. We also used the reference gene Rpl16a as an additional control in each analysis.

## Discussion

Calcium ions are crucial for the contraction of all types of muscles. After influx into the cytoplasm, they interact with myofilaments and ultimately allow for interaction between myosin and actin filaments, and thus for muscle contraction. Since they are a trigger and an executor of muscle contractions, their concentration in the sarcoplasm must be strictly regulated. In striated muscles, cell membrane depolarization is a signal that initiates the cascade responsible for muscle contraction. Changes in the cell membrane potential activate and open the L-type calcium channels. Then, the local increase in Ca^2+^ concentration activates the ryanodine receptor, a sarcoplasmic calcium channel, which releases the next portion of calcium ions into the cytoplasm, which interacts with myofilaments.

L-type calcium channels are responsible for the initiation of the Ca^2+^ transition into cells, among other phenomena. The α1 subunit of this channel forms a selective pore and determines its electrophysiological and pharmacological properties. For instance, the channel possesses sites sensitive to changes in cell membrane potential and sites for docking of many bioactive compounds, such as the calcium channel blockers dihydropyridine or verapamil (Bodi et al. 2005). Thus, the observed decrease in heart contraction frequency, or even arrest of heart contraction after application of verapamil solutions, pharmacologically confirms the presence of the L-type calcium channel in the myocardium of the tested insect. Our results are consistent with the observations of Gu and Singh (1995), who showed that verapamil decreased contractile activity in the heart of *D. melanogaster* larvae. However, Johnson et al. (1998) showed that this blocker did not affect *D. melanogaster* pupa heart activity. However, both studies mentioned above were conducted under *in vivo* conditions; thus, the final effect could be modified through the influence of verapamil on the L-calcium channels also present in other tissues, especially in the nervous system. The identification of the transcript for the α1 subunit of the L-type calcium channel in the myocardium of the beetle *T. molitor* confirms that the observed cardioinhibitory effects of verapamil in the heart of *T. molitor* result from the blocking of L-type calcium channels by verapamil. Our studies also showed that two forms of the transcript are present in the myocardium, brain and entire body. The presence of α1-subunits in insect myocardial cells was also confirmed in *D. melanogaster* (Lam et al. 2018; Limpitikul et al. 2018) and in *M. domestica* (Grabner et al. 1994), but only one form of the transcript was indicated.

Similar to verapamil, cardioinhibitory activity was also observed for SGAs. When applied to a semi-isolated insect heart, SGAs caused a reversible decrease in heart contraction frequency. These cardioinhibitory properties of SGAs have previously been shown by studies conducted by Ventrella et al. (2015) and Marciniak et al. (2019). Ventrella et al. (2015) demonstrated that α-chaconine and α-solanine decreased the frequency of insect myocardium contractions in the adult beetle *Zophobas atratus*, both under *in vitro* and *in vivo* conditions (Ventrella et al. 2015). In turn, Marciniak et al. (2019) confirmed the cardioactivity of α-chaconine and α-solanine in pupae of *T. molitor in vivo,* also showing that SGA cardioactivity significantly depends on the phase of the circadian rhythm of heart activity. Depending on the phase, SGAs might stimulate or inhibit heart contraction. Furthermore, they showed that SGAs affect the frequency of heart contraction and the duration of phases of heart activity (anterograde, retrograde and diastase phases) (Marciniak et al. 2019). The cardioactivity of SGAs is likely a result of their ability to alter the homeostasis of calcium, potassium and sodium ions across a cell membrane. It was shown that α-chaconine and α-solanine change the intracellular concentration of free Ca^2+^ ions in several types of cells (Toyoda et al. 1991; Blankemeyer et al. 1995, 1997, 1998). The intracellular Ca^2+^ concentration increased in NG 108-15 hybrid cells (mouse neuroblastoma x rat glioma) when treated with α-chaconine or α-solanine, and the changes showed some dose dependency. It should also be noted that the Ca^2+^ influx evoked by α-chaconine was not prevented by mono- and divalent metal ions or by inhibitors of Ca^2+^ transport across membranes, such as voltage-operated channel antagonists, muscarinic and nicotinic antagonists, or Na^+^ and K^+^ channel blockers (Toyoda et al. 1991). The cardioinhibitory activity of SGAs seems to be a paradox if one considers their ability to increase intracellular calcium levels. Because of that, they should rather act in cardiostimulatory fashion.

Nevertheless, SGAs affect not only passive but also active ion transport. For example, α-solanine was found to inhibit active calcium transport in a rat duodenum (Michalska et al. 1985), and α-chaconine or α-solanine decreased the transepithelial active transport of sodium ions in frog skin (Blankemeyer et al. 1995, 1997). Moreover, the above data suggest that SGAs might act both in channels ion transporter-dependent and ion transporter-independent ways. Moreover, SGAs interact with cell membrane cholesterol. α-Solanine and α-chaconine can form tubular structures within cell membrane monolayers in artificial phospholipid vesicles, increasing the permeability of membrane structures for different ions (Keukens et al. 1995, 1996). Thus, the effects of SGAs on ion balance might change the cell membrane potential and thus modulate the activity of excitable cells, including myocardial cells. Blankemeyer et al. (1998) showed that solasonine and solamargine decrease the cell membrane potential (hyperpolarization) in frog embryo cells. If the same occurs in myocardial cells, it could explain the cardioinhibitory properties of SGAs.

What draws attention is differences between kinetic of α-solanine and α-chaconine at the highest tested concentration. α-Solanine reaches the maximal cardioinhibitory effect between 6 and 7 min and further application did not change the heart contraction frequency, thus the plateau was noticed between 7 and 13 min of registration. Whereas, in case of α-chaconine, during the entire period of application, an increasing cardioinhibitory effect was observed, and for that glycoalkaloid we did not observed plateau. The explanation of this phenomenon might be a saturation of target/targets (receptors/cell membrane components or others) with which the tested glycoalkaloids interact. Obtained data suggest that α-solanine might reach the point of saturation earlier than α-chaconine. It is possible, that for longer time of application used for α-chaconine, the plateau would be also observed. On the other hand, observed difference can result from different mode of action of both glycoalkaloids and that they interact with various targets. Similar differences were shown by Bielawski (1990) for detergents inducing haemolysis.

In our studies, the simultaneous application of verapamil and SGAs onto the heart resulted in changes in the effects caused by SGAs when compared to the protocol in which the compounds were applied separately. The modifying action of SGAs on verapamil activity differed slightly depending on the methods of application of the tested glycoalkaloids (experimental variants A and B). However, in general, the tested glycoalkaloids weakened the cardioinhibitory effect induced by verapamil. Considering that both verapamil and SGAs manifest cardioinhibitory activity, the changes in their activity suggest that they work antagonistically (Table 1).

To explain this phenomenon, it is important that verapamil binds to L-type calcium channels in a voltage-dependent manner, meaning that affinity is increased as the cell membrane potential is reduced and with an excessive depolarizing stimulus (Kanaya et al. 1983; Sanguinetti and Kass 1984). SGAs change the cell membrane potential, e.g., by increasing intracellular concentrations of Ca^2+^ or Na^+^ ions. In that case, they might also decrease the affinity of verapamil to calcium channels, and in that way, the cardioinhibitory effect of this agent diminishes. Gee et al. (1996) showed that SGAs caused partial depolarization of the cell membrane of rat epithelial cells probably by changing the cell membrane permeability for Na^+^; thus, the efficiency of verapamil blocking of L-type calcium channels in the presence of SGAs could decrease. On the other hand, Blankemeyer et al. (1998) demonstrated that α-solamargine and α-solasonine decrease the cell membrane potential, causing hyperpolarization of *Xenopus laevis* frog embryo cells. It should be kept in mind that verapamil affects not only calcium channels, but also rapid delayed rectifier potassium channels (e.g., the K_v_11.1 channel), which mediate the cardiac I_Kr_ current that acts as an essential determinant of action potential repolarization in the human ventricle; thus, their inhibition hinders the reconstitution of resting potential (Zhang et al. 1999). Therefore, hypothetically, if SGAs cause hyperpolarization by efflux of K^+^ from the cells, they can abolish the effect of verapamil caused by inhibition of the K_v_11.1 channel and simultaneously cause cardioinhibition in the absence of verapamil. More studies are needed to confirm this hypothesis.

The other explanation for the modification of verapamil activity by SGAs involves the ability of SGAs to increase the intracellular calcium concentration (Toyoda et al. 1991), which might partially counteract the cardioinhibitory effects caused by verapamil. Moreover, it should be noted that SGAs, by incorporation into the cell membrane and interaction with cholesterol (Keukens et al. 1995, 1996), might not only change the permeability of the cell membrane but can also affect the conformation of many cell membrane proteins, including calcium channels as well as other channels.

Another possible site of interaction between verapamil and SGAs is P-glycoprotein, a membrane transporter responsible for removing xenobiotics from cells. Verapamil and SGAs inhibit the activity of this protein (Summers et al. 2004; Li et al. 2011). The simultaneous interaction between verapamil and SGAs on P-glycoprotein might change their pharmacokinetic properties.

More studies are needed to better understand and explain the interactions between SGAs and verapamil. For example, can SGAs interact with the ryanodine receptor, another crucial channel for myocardium contraction?

## Conclusions

The results demonstrate that the cardioactive properties of verapamil are strongly affected by glycoalkaloids α-solanine and α-chaconine. They act antagonistically to verapamil and decrease its action efficiency. They cause not only a change in maximal cardioinhibitory effect evoked by verapamil, but also change the dynamics of verapamil action as t_50_ and recovery time. Our studies demonstrated that the activity of popular drugs such as verapamil could be modified by glycoalkaloids commonly present in many food products. This shows that attention to the composition of the daily diet during therapy with various drugs is very important. Of course, more detailed research is needed in that case.
